# Language and Cognitive Development in Bimodal Bilingual Deaf Children in Hearing Families: Three Case Studies

**DOI:** 10.3390/bs15081124

**Published:** 2025-08-19

**Authors:** Diane Lillo-Martin, Deborah Chen Pichler, Elaine Gale

**Affiliations:** 1 Department of Linguistics, College of Liberal Arts & Sciences, University of Connecticut, Storrs, CT 06269, USA; 2 Department of Linguistics, School of Language, Education, and Culture, Gallaudet University, Washington, DC 20002, USA; deborah.pichler@gallaudet.edu; 3 Department of Special Education, School of Education, Hunter College, City University of New York, New York, NY 10065, USA; egale@hunter.cuny.edu

**Keywords:** deaf and hard-of-hearing children, American Sign Language, bimodal bilingualism, language development, cognitive development

## Abstract

We investigated aspects of language and cognitive development in three bimodal bilingual deaf children in hearing families. Some previous research finds cognitive delays for deaf children, which may be due to an early lack of access to language input. Studies of children having strong early language access through parental use of American Sign Language (ASL) support the hypothesis that language delays are behind such cognitive delays. We ask whether hearing parents who are novice learners of ASL provide sufficient support for early language and cognitive development. The three case studies in this report used both ASL and English, with support for ASL development provided by our ASL specialist. We assessed the children’s general cognition, executive function, ASL vocabulary, English vocabulary, and overall language ability in both ASL and English. We found strong language development outcomes and correspondingly, age-appropriate cognitive development. These results are consistent with the conclusion that novice signer parents can support their children’s development as ASL-English bilinguals, establishing a strong foundation for further cognitive and linguistic growth.

## 1. Introduction

Under typical circumstances, language and cognitive development go hand-in-hand (Deák, 2014). For deaf and hard-of-hearing (DHH) children, language development varies according to many factors, including the age at which language access is available. For some DHH children, the first accessible language is a visual language, such as American Sign Language (ASL). For others, given hearing technologies such as hearing aids and/or cochlear implants, the first language will be a spoken language, such as English. In either case, the age of access and the quality of input may vary. Thus, differences in early language exposure may lead to potential delays in both language and cognitive development (Bouzaher et al., 2024; Ching et al., 2019).

Given these factors, it is not surprising that some studies report substantial differences in cognitive development of DHH children compared to typically hearing (TH) children (Bouzaher et al., 2024). Since many aspects of cognitive development typically go hand-in-hand with linguistic development, when children experience language delays, associated delays in certain aspects of cognitive development can also be expected (Morgan et al., 2020). Yet, many studies of cognitive development do not take into account children’s language levels (in one or multiple languages), or even use the child’s preferred accessible language for assessment (Mayberry, 2002). Consideration of these variables yields results that more accurately reflect the child’s capabilities. Even still, it must be noted that frequently, assessments use normative samples from monolingual children who have typical hearing; such comparisons are often inappropriate and resulting findings should be interpreted with great caution (Ortiz & Cehelyk, 2024).

In this article, we report language and cognitive development findings from three case studies of DHH children. The children’s situation is of interest because of their linguistic environment. Their parents have typical hearing and did not know ASL before their children were born. However, while their children were infants, they chose to begin to learn ASL and provide their children with exposure to this language. In addition, they chose to provide technological support to their children’s hearing through bilateral cochlear implantation (CI). With appropriate spoken language therapy, the children were also developing spoken English. Under such circumstances, we are in a position to report on both language and cognitive development for emerging bimodal bilinguals.

In the next sections, we provide relevant background information about language (Section 2) and cognitive (Section 3) development for DHH children. Then we go on to describe the current research project.

## 2. Background: Language Development in Deaf and Hard-of-Hearing Children

About 5% of DHH children are born to deaf parents (Mitchell & Karchmer, 2004), who may use a natural sign language, such as American Sign Language, providing their children with full accessible linguistic input from birth. First language acquisition of a sign language under such circumstances proceeds in a typical manner, comparable to the development of a spoken language under similar circumstances (Chen Pichler et al., 2018; Lillo-Martin & Henner, 2021).

In some cases, DHH children with deaf parents also use hearing technology such as CIs to support their development of a spoken language (see chapters in Paludneviciene & Leigh, 2011). Studies of the development of bimodal bilingualism (two languages in two modalities, sign and speech) have generally found parallels to unimodal bilingualism. With sufficient access to both languages, children become bilingual; like other bilinguals, consequences such as cross-language influence and code-switching (or code-blending) can be observed. Since the population of DHH children of deaf families using CIs and sign language is quite small, there are few reports, and they involve very few participants. See Cassandro et al. (2003) for a study of one child learning Italian Sign Language and Italian; Hassanzadeh (2012) for a study of 7 children learning Persian and Persian Sign Language; Davidson et al. (2014) for a study of 5 children, and Goodwin and Lillo-Martin (2023) for a study of 6 children learning ASL and English.

The remaining 95% of DHH children are born to hearing parents, who typically have no knowledge of a sign language before their child is identified. Inevitably, there is a high risk for delayed access to linguistic input for these children, because it takes time for confirmation of diagnosis and for parents to decide on how they will communicate with their child and act on this decision. It is recommended that infants receive hearing screening by 1 month of age, audiological diagnosis by 2 months, and enrollment in early intervention by 3 months (The Joint Committee on Infant Hearing, 2019), although these goals are not always met. Once a diagnosis is confirmed, parents face many choices regarding their next steps. Among these is the choice of language access: will the child receive hearing aids and/or cochlear implants to increase access to sound; will the child receive input in a natural sign language such as ASL; or both? Until such time as one or both of these steps are taken, the child is likely to experience some degree of language deprivation due to lack of accessible linguistic input (W. C. Hall et al., 2017). If the parents choose to use spoken language exclusively, some months or even years may pass while the child is fitted with appropriate hearing technology and provided with speech training. If the parents choose to expose their child to a natural sign language, they must find appropriate resources to learn the language and ensure adequate levels of exposure for their child. In many cases, parents who choose sign language also pursue spoken language, necessitating both sets of steps.

During the preschool years, spoken language development for DHH children using CIs may reach levels seen in typically hearing peers, but the outcomes are highly variable. While some children have good spoken language outcomes, a large proportion of DHH children achieve spoken language levels that are below expectations (Antia et al., 2020; Ching et al., 2017, 2025; Dettman et al., 2021; Geers et al., 2017b; Karltorp et al., 2020; Lederberg et al., 2019; Niparko et al., 2010).

For example, the histogram of Global Language Scores reported by Ching et al. (2017, p. 7) shows that roughly 50% of participants achieved a score of 85 or higher, that is, within one standard deviation of the mean for a normal population or higher.^1^ While performance within one standard deviation of the mean is generally not considered clinically significant, in a normal distribution only about 16% of the population would score more than one standard deviation below the mean. A situation in which half of participants score in this range would be considered cause for concern (see M. L. Hall, 2020 for extended discussion on this point).

Recent research has demonstrated that several factors are typically associated with higher or lower outcomes. Presence of disabilities is associated with lower outcomes, and more severe-profound hearing levels are associated with lower outcomes. On the other hand, higher maternal education and earlier age at hearing device fitting are associated with higher outcomes (Cupples et al., 2018).

Thus, despite recent advances, it is not currently possible to predict how successful a given child’s outcomes will be. Accordingly, it would seem prudent to begin using an accessible sign language with young DHH children as early as possible, so that the child can establish a solid linguistic foundation on which cognitive, academic, and additional language skills can be built (Humphries et al., 2022). However, parents are often directed instead towards interventions that focus on developing spoken language exclusively (Jones & Roberts, 2024).

Arguments for an “oral only” approach often rest on widespread claims about signing that sound logical, but are in fact inaccurate (Lillo-Martin et al., 2023). For example, it may seem unlikely that parents would be able to learn a sign language fast enough or fluently enough to be able to support their child’s development. Geers et al. (2017a, p. 4) put it clearly: “the majority of hearing parents typically lack proficiency in ASL and, therefore, cannot provide a language-rich environment in both ASL and spoken English.” One of our goals is to address this issue by evaluating parents’ development of ASL. We also examine language and cognitive development by their deaf children, in both of their languages. Initial evidence that parents can in fact learn enough to support their children’s development comes from studies by Caselli et al. (2021), Finton et al. (2024), and Pontecorvo et al. (2023).

Another common misconception is that unlike spoken languages, sign languages have no critical periods and can be learned equally well at any stage of life, after spoken English is firmly established. However, it is studies of deaf signers that actually provide the strongest evidence for the critical period hypothesis: when language access is delayed, lifelong effects can be observed (see Mayberry & Kluender, 2018 for a review of this evidence).

In the face of such advice against early sign language exposure, on top of the effort required of parents who pursue this path, it is not surprising that only a minority of hearing parents ultimately choose to sign with their DHH child.^2^

When a family does decide to pursue a sign language with their DHH child, what does the child’s language and cognitive development look like? A handful of studies have begun to address this question, among which is the current project. To date, few details are available about the quality or quantity of sign language input that hearing sign language learners provide to their DHH children (M. L. Hall, 2020; M. L. Hall et al., 2019), but it appears that a growing subset of hearing parents are highly motivated to learn a sign language (Chen Pichler, 2021; Lieberman et al., 2024) and are doing so well (Pontecorvo et al., 2024).

Caselli et al. (2021) measured the ASL vocabularies of deaf children from hearing families, who received exposure to signing by the age of 36 months. The children who were exposed to ASL by 6 months of age scored at the same level as the control group of deaf children with deaf, signing parents. Importantly, early ASL development also benefits spoken language vocabulary development; Pontecorvo et al. (2023) reported that children’s English vocabulary size was higher for DHH children whose ASL vocabulary was higher. Early sign language input appears to confer advantages even when provided short-term by non-native signers. Delcenserie et al. (2024) studied DHH children (5;01–7;01 [years;months]) exposed to non-native LSQ (Quebec Sign Language) input from their hearing parents before receiving their CIs and found positive effects on their phonological short term and working memory; children whose parents continued to use LSQ for a few months following CI activation showed additional benefits to general language development.

Though still few in number, the studies cited above provide motivation for the current project. We are tracking language and cognitive development in deaf children whose hearing parents have chosen to incorporate ASL as one of their family’s languages, studying not only the linguistic and cognitive development of the deaf children, but also the second language (L2) sign language development of their hearing parents. Our project is described in Section 4, following which we describe results from three case studies: the first three families who enrolled in our longitudinal study for over a year. Before that, we provide additional background information regarding cognitive development in DHH children.

## 3. Background: Cognitive Development in Deaf and Hard-of-Hearing Children

Parents are frequently concerned that their DHH child may suffer delays and/or deficits in cognitive development compared to TH age-peers, a view which has sometimes been supported by research (for discussion, see Vernon, 2005). Indeed, a recent systematic review found that DHH children “may perform lower than their age-matched normal hearing peers on IQ Testing across a battery of IQ testing modalities” (Bouzaher et al., 2024). The review authors came to this conclusion based on mixed evidence across various studies, with some showing no significant differences in scores between DHH and TH children, while others reported just such a difference.

When researchers distinguish between (so-called^3^) non-verbal and verbal measures, it is often the case that DHH and TH children do not differ on the non-verbal measures (e.g., Braden, 1994; Phillips et al., 2014); in fact, some studies find enhanced performance by deaf children who come from deaf, signing families, as compared to TH children (see review in Mayberry, 2002). In contrast, scores of DHH and TH children often differ on verbal measures. This can be seen for children with high spoken language skills tested using spoken language, and for signing children tested in a nonstandard way using a sign language (Mayberry, 2002). Akamatsu et al. (2008, p. 143) summarized these findings as “the reliable, although not necessarily valid, finding of deaf people’s VIQ [verbal IQ] being approximately one standard deviation below that of the hearing population.” Among the reasons casting doubt on these results are the fact that these studies employ norms from monolingual, typically hearing, English-speaking individuals; that signed versions of the measures are administered in a nonstandard method; and that these studies generally fail to account for many potentially impactful variables in the deaf population (Maller, 2003).

Turning to social–emotional development, a review of US National Health Interview Survey (NHIS) results (W. C. Hall et al., 2018) found that respondents with a deaf child were more likely to respond ‘somewhat true’ or ‘not true’ when asked if their child was well-behaved, compared to respondents with hearing children. A systematic review found that ratings of DHH children having emotional and behavioral difficulties were ‘about a quarter to a third of a standard deviation higher than [for] hearing children’ (Stevenson et al., 2015). These studies did not report information about children’s families or language development. However, Stevenson et al. (2010) conducted a previous study of 120 DHH children in England that found overall higher levels of behavior problems in comparison to hearing children; these problems were highest among DHH children with the least developed language abilities. Similarly, in a study of 334 DHH children in Denmark, Dammeyer (2010) reported a much higher prevalence of psychosocial difficulties for the deaf children overall, in comparison to TH children. However, no such higher level of difficulties was found for children with good existing language skills, whether spoken or signed.

Among the many areas of cognitive development that have received specific attention, executive function (EF) has been particularly widely studied in DHH individuals. EF is a cover term for a range of cognitive abilities that contribute to general control and coordination of behaviors. As described by Goodwin et al. (2022, p. 209), EF refers to “behaviors that allow an individual to overcome more automatic or established responses to achieve a goal.” One commonly-used measure of EF is the Behavioral Rating Inventory of Executive Function (BRIEF; Gioia et al., 2000; or BRIEF-Preschool for children ages 3–5, Gioia et al., 2003) which uses parent report to assess three EF components: working memory, inhibition, and shifting. The BRIEF asks parents to what degree certain behaviors are problematic in their children. These behaviors are allocated to five distinct scales that contribute to three Summary Indices and a Global Executive Composite score, with a mean of 50 and potential clinical significance for scores above 65 (as higher scores indicate greater problematic behaviors).

Several previous research studies indicate poorer results on EF measures for DHH children (Beer et al., 2011; Jamsek et al., 2022; Kronenberger et al., 2014; Pisoni et al., 2010). In some studies, group differences on BRIEF scores are no longer significant if language abilities are controlled for (Beer et al., 2014). Such a result may be expected, as measures of EF and language are often highly correlated (Shokrkon & Nicoladis, 2022). M. L. Hall et al. (2017) asked deaf parents of DHH children (ages 5;01–12;10) to complete the BRIEF, and compared their results to BRIEF inventories collected from parents of TH children. Neither group’s mean exceeded the expected value of 50 on any subscale or summary index, meaning that problematic behaviors were reported to be no more than those in the norming population. Goodwin et al. (2022) used the BRIEF-P with four groups: Typical Hearing, Deaf with Early ASL, Deaf with Later English, and Deaf with Later ASL (ages 3;01–7;07). They found no significant differences between hearing and deaf participants, but age at language exposure (whether in speech or in sign) was a significant predictor overall and on several subscales.

Taken together, the literature reviewed here confirms that there is a relationship between language and aspects of cognitive development for DHH children. In most of these studies, children with early sign language exposure had deaf, signing parents. Today however, more hearing parents are learning to sign with their DHH children at earlier and earlier ages. Studies have begun to examine language development under such circumstances, but more research is needed. This need led to our current research, the Family ASL project, which we describe in the following section.

## 4. The Family ASL Project

The goals of the Family ASL project are to investigate the development of DHH children between 2 and 5 years old whose hearing parents have decided to use ASL with them. This study is unusual in that we also carefully track and document the development of ASL as a second language by the children’s hearing parents. There are both cross-sectional and longitudinal components of the study; here we discuss the longitudinal component in which the three families of the current report were participants. This component includes weekly data submission, services of an ASL specialist, and sets of tasks (assessments) administered over the course of more than a year.

After going through screening and giving informed consent, families were scheduled for the first set of tasks. Under the guidance of a member of the research team, families started recording language samples. Families were instructed to record language samples of approximately 30 min using their typical form of communication while engaging with their child in common activities such as playing with toys, looking at books, or having a snack.

At this time, families also began one-on-one meetings with the ASL specialist. The ASL specialist is a deaf signer with many years of experience teaching ASL. The sessions involved teaching aspects of ASL vocabulary and grammar, targeted towards the needs of each individual family. Meetings with the ASL specialist were scheduled in 6-week blocks (6 weeks on, followed by 6 weeks off) over the course of 48 weeks.

**Figure 1:**
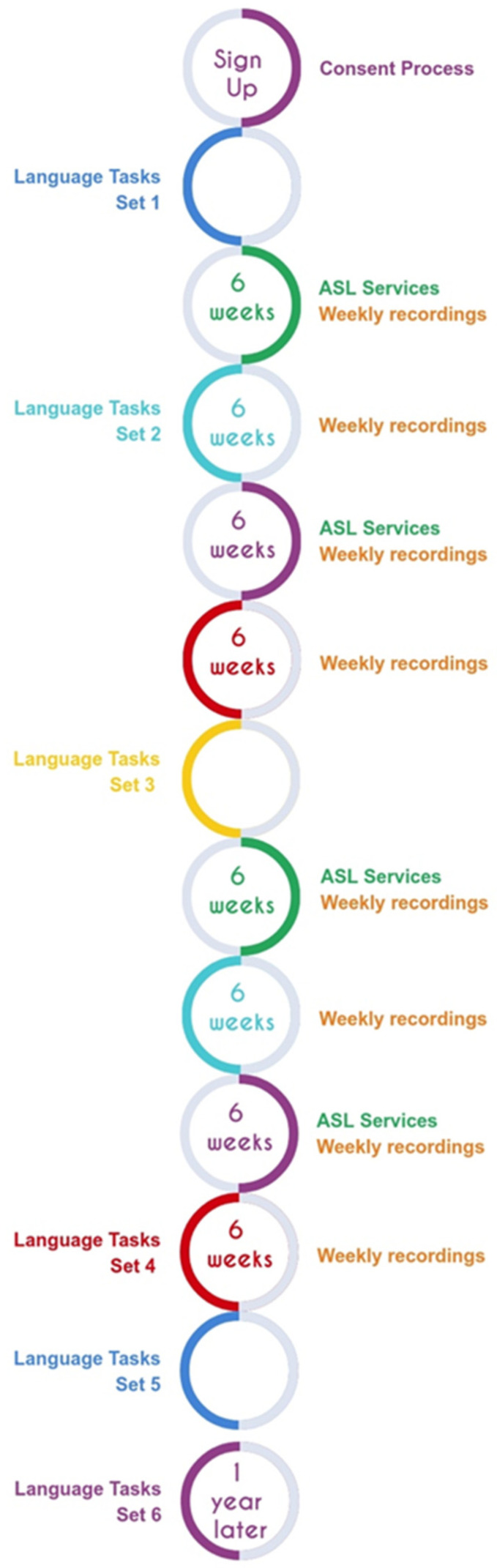
Study timeline.

The language and cognitive assessments were scheduled during ASL services off weeks, to facilitate participation. A final follow-up set of tasks was scheduled for 6–12 months later, as long as the child was at least 4 years of age. (See Figure 1 for Study Timeline). Using this design, we were able to administer most tasks described in the present paper three times over the course of the child’s participation. Since the intervals between administrations varied, the child’s age at each measurement is included in the tables of results.

All assessments were administered remotely. A research assistant met with the child (where applicable) and their participating parent over Zoom. Each child direct assessment lasted approximately 20 to 30 min; parent reports required up to one hour. The tasks were administered by members of the team using spoken English, or, for ASL tasks, using a blend of spoken English and ASL. The Zoom sessions were recorded so that scoring or checking could be performed off-line.

The aims of the current report are to present a subset of results from the first three participants who completed the full set of tasks in the longitudinal study. These results include two non-language assessments and two language assessments for ASL and for English. Given our review of the literature, we expect that if language assessments (ASL and/or English) are within or close to expectations for age, then the non-language assessments should also display age-appropriate levels.

## 5. The Case Studies

The participants are three deaf children of hearing parents, who participated in the Family ASL project over a period of one year, with some follow-up observations occurring 8 to 16 months after completion of the longitudinal study. The children were between the ages of 2 and 3 when they joined the study. These three participants were selected for the current report because they were the first to complete the longitudinal study. More comprehensive reports on the project overall are in preparation. Parents had previously and independently chosen what hearing technology to use, and they had elected to use sign language with their child. A summary of the children’s characteristics is given in Table 1, followed by case reports of the children’s relevant hearing and linguistic background before joining the study. The case reports are primarily based on our administration of the Language Access Profile Tool (LAPT; M. L. Hall & De Anda, 2021, 2022).

Ellie was born profoundly deaf in both ears. She used bilateral hearing aids from 7 to 9 months of age, but discontinued use. She received bilateral cochlear implants at the age of 19 months, with activation at 20 months; she is reported to use the devices all day. When Ellie was 2 months old, her family started learning ASL and using it with her. She received ASL support from a local deaf community resource center. Her mother reports using speech and signs together for a majority of the time prior to joining our study.

Nayla experienced sensorineural hearing loss at birth, progressively increasing to profound levels between birth and the age of 3 months. She used hearing aids from the age of 3 months, but due to various issues, it is unknown whether she had access to sound until the age of 9 months, when she began seeing a new provider. At 26 months, she received bilateral cochlear implants which were activated shortly thereafter. At enrollment in our study, her mother reported that she uses the devices about 4–6 h per day. Although the family was discouraged from using ASL, when Nayla was 8 months of age her family started taking ASL courses. Her mother reported using a combination of speech and signs with her; she also had a Deaf mentor and ASL tutor from whom she received ASL exposure.

Haven was born with congenital Cytomegalovirus (CMV); she was profoundly deaf at birth in her right ear and experienced a progressive hearing loss from mild to profound by age 9–10 months in her left ear. Haven received bilateral cochlear implants at the age of 10 months, with activation at 12 months; she uses the devices all day. Her mother took ASL courses starting when Haven was 4 months old, but she also reported that Haven’s exposure to ASL was limited. To increase Haven’s signed exposure, her mother enrolled her in a split preschool program that was half mainstream (using spoken language only) and half in ASL.

Parental sign. The questions of how well the hearing parents of these children acquire ASL as a second language and use it as a home language are of paramount importance to our project, given the obvious role of parental language input for children’s early cognitive and linguistic development. An in-depth discussion of the parents’ ASL proficiency lies outside the scope of the current article, but we will touch on a few points related to parents’ L2 ASL, to illustrate the variation in signed input that we observe across families in our project.

None of the parents of Ellie, Nayla or Haven knew ASL at the time their DHH child was born. After their child was diagnosed, parents’ ASL learning experiences varied quite a bit. They received home visits from Deaf adults, used digital resources for learning ASL, and took ASL classes designed for hearing parents. The mothers of Haven and Ellie also sought out formal ASL courses at community colleges or universities. By the time they joined our study, Ellie’s mother self-evaluated her ASL proficiency as “fairly fluent,” while Nayla’s and Haven’s mothers reported that they could express most of what they wanted to say in ASL, but with difficulty.

In addition to soliciting parents’ self-perceptions of their ASL proficiency, we also performed our own evaluations, based on ASL data that we collected from participants over the course of the project. While a more detailed presentation of parental sign development is separately in preparation, here we provide partial information regarding parents’ ASL vocabulary. One parent for each participating child (in the present cases, the mother) was administered a 100-sign version of the American Sign Language Communicative Development Inventory (ASL-CDI) (Caselli et al., 2020) three times over the course of the study (see Section 6.3 for a description of this assessment). Results of this assessment, expressed as number of items out of 100, are provided in Table 2, along with the age of each child at the time of the mother’s assessment. These results reflect linguistic growth, albeit with individual variation.

**Figure 2:**
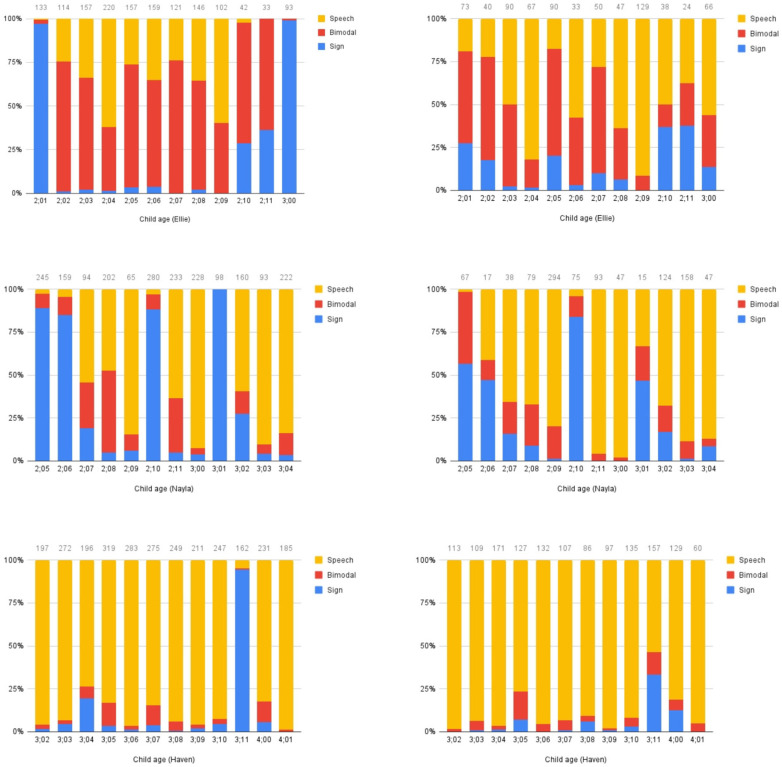
Modality counts. Proportion of utterances produced in speech only, bimodal, or sign only. Left column shows adults; right column shows children. Top row Ellie, middle row Nayla, bottom row Haven. Numbers above bars indicate total number of utterances coded.

Modality. We assessed the children’s and parents’ communicative modality through analysis of language samples recorded throughout the period of the study. We analyzed 10 or 15 min monthly samples of the parent and child interacting. For each sample, we determined whether any utterances were produced using (a) speech only; (b) a combination of sign and speech (bimodal); or (c) sign only. Results are presented in Figure 2.

General patterns can be observed from these plots. Ellie’s mother produces a large amount of code-blending, and some speech-only utterances. Once in a while, she produces a large stretch of sign-only utterances, as in the first and last samples. Ellie herself produces a mixture of bimodal and speech-only utterances, with a smaller proportion of sign-only utterances. Nayla’s mother primarily uses sign-only or speech-only utterances, with a small proportion of bimodal utterances. Some sessions have a high proportion of sign-only utterances, while others have relatively low proportions of sign-only content. Nayla produces a lot of speech-only utterances, with sign-only utterances in fewer sessions, and little blending after the first session. Haven’s mother and Haven herself produce almost exclusively speech-only utterances in these sessions, with the exception of one session (at 3;11), during which her mother produced a high proportion of sign-only utterances, and Haven also used a higher than normal proportion of sign-only and bimodal utterances. The variability in modality choices we observe may originate from the mothers, may indicate that mothers are responding to their child’s preferences, or may be a reflection of a two-way interaction where each interlocutor responds to the choices of the other (see Lillo-Martin et al., 2014 for an extended assessment of modality choices made by bimodal bilingual children and their interlocutors).

## 6. Materials

We present first the two non-language assessments, then the two language assessments (vocabulary and overall language).

### 6.1. Task 1: Screening

We administered the Cognitive and Social–Emotional subtests of the Developmental Assessment of Young Children-2 (DAYC-2; Voress et al., 2012). This assessment is conducted as a structured interview with the child’s parent, who answers questions about the child. The researcher starts at an age-appropriate point of the instrument and asks whether the child exhibits that behavior. For example, one of the items for children in the 24–35-month age group is “asks for assistance when having difficulty”. Parents reply by scoring a 1 (yes) or 0 (no). Ceiling is reached when three consecutive items receive a score of 0. Raw scores are converted into normative scores, based on the instrument’s normative sample of TH children.

### 6.2. Task 2: Executive Function

We administered the Behavioral Rating Inventory of Executive Function-Preschool Version (BRIEF-P; Gioia et al., 2003). Parents are asked how often each behavior has been a problem over the past 6 months (possible responses: never, sometimes, or often.) Parent responses are converted into T-scores with a normative mean of 50. A score of 65 or greater indicates potential clinical significance.

Questions on the BRIEF-P contribute information about 5 clinical scales. The Inhibit and Emotional Control scales are combined into an Inhibitory Self-Control Index (ISCI); the Shift and Emotional Control scales are combined into a Flexibility Index (FI); and the Working Memory and Plan/Organize scales are combined into an Emergent Metacognition Index (EMI). All five scales contribute to the Global Executive Composite (GEC).

### 6.3. Task 3: Vocabulary

We assessed participants’ vocabulary in ASL and in English, using similar parent report measures described below.

ASL Materials. With the cooperation of Naomi Caselli, we developed a 100-item version of the American Sign Language Communicative Development Inventory (ASL-CDI; Caselli et al., 2020). We created two forms with different items; both forms used numbers of words in different semantic categories proportional to the full version. We used Form A for Time 1 and Time 3, and Form B for Time 2. As in the full version, we showed parents a video recording of individual signs; we asked them to report on both their child’s knowledge and their own knowledge of each sign. For each item, the following choices were made available: (i) understands; (ii) understands and signs; (iii) uses a different sign for this; (iv) does not know this sign; and (v) skip/no answer. We report children’s receptive scores (any signs that were marked as (i), (ii), or (iii)) and their expressive scores ((ii) or (iii)). The scores of our participants can be compared to the results provided by Caselli et al. (2020) for similar-aged deaf children with signing, deaf parents.

English materials. We administered a modified version of the 100-item short form version of the English MacArthur-Bates Communicative Development Inventory (CDI; Fenson et al., 2000). Parents completed a form asking them to indicate for each word whether their child understands and says the word (expressive only). The instructions told parents to mark the words even if their child does not pronounce the word exactly right or uses a different word with the same meaning. We used Form A for administration at Time 1 and Time 3, and Form B for administration at Time 2. Scores are reported as the number of known words out of 100 and compared to the percentile bands indicated in the MacArthur Bates manual (Marchman et al., 2023) for typically hearing monolingual children. Note that the English CDI manual provides percentile scores only up until the age of 30 months.

### 6.4. Task 4: Overall Language

To measure overall language development in ASL and English, we used two assessments that are frequently employed with DHH children: the Receptive Skills Test (ASL-RST; Enns et al., 2013) for ASL and the Clinical Evaluation of Language Fundamentals Preschool-3 (CELF-P3; Wiig et al., 2020) for English. Both assessments were administered once per child, in the follow-up sessions that took place after the participants turned 4 years old.

ASL materials. The ASL-RST (Allen & Enns, 2013; Enns et al., 2013; Enns & Herman, 2011) is an ASL comprehension test adapted from the BSL Receptive Skills Test (Herman et al., 1999). We administered this assessment online through a portal for the administration of ASL assessments maintained by the Gallaudet University VL2 research group. The child participant views a signed sentence and selects one picture out of 4 that matches the sentence. Enns et al. (2013) provide standard scores based on a standardization study with 203 participants, of whom 77 received ASL input from birth, while the remaining 126 received input before the age of 3.

English materials. The CELF P-3 (Wiig et al., 2020) is an omnibus language test that measures a child’s overall spoken English skills. We administered the three subtests that make up the “Core Language” score: Sentence Comprehension, Word Structure, and Expressive Vocabulary. In the ‘sentence comprehension’ subtest, the child is asked to point to the picture that corresponds to a spoken sentence. To accommodate online administration, the child’s parent reported aloud to the experimenters which picture (labeled a, b, c, or d) the child pointed to.

## 7. Results

In this section we provide the numerical results of each assessment with a simple summary. The results are given in the order of presentation of materials in the previous section: non-language tasks, vocabulary tasks, and overall language tasks. Deeper interpretation and discussion of results is held for the Discussion Section.

### 7.1. Task 1: Screening

Results from administration of the DAYC-2 are provided in Table 3. Scores are expressed as standard scores based on the hearing, monolingual normative sample. Performance in the range of 85–115 is interpreted as within one standard deviation of the mean of the normative sample. All scores of the three participants fall within this range.

### 7.2. Task 2: Executive Function

Results from administration of the BRIEF-P are provided in Table 4. T-scores have a normative mean of 50, with higher scores indicating greater problematic behavior; a score of 65 or greater indicates potential clinical significance.

### 7.3. Task 3: Vocabulary

Results from administration of the ASL-CDI (100-item version) are provided in Table 5. Scores are given as number of items out of 100. An asterisk indicates that a given score is proportionally at or above the mean for participants of the same age in the full version of the assessment.

Results from administration of the English CDI (100-item version) are provided in Table 6. Scores are given as number of items out of 100. Interpretation of results is held until the Discussion section, as norms for monolingual hearing children are only available up to the age of 30 months.

### 7.4. Task 4: Overall Language

Results from administration of the ASL-RST are provided in Table 7. Both raw accuracy and standard scores are provided.

Results from administration of the CELF-P3 Core Language subscale are provided in Table 8. Scores are given as standard scores in comparison to the hearing, monolingual normative sample.

## 8. Discussion

We begin by interpreting the scores of the three case studies on the non-language tasks, and then we present the language tasks. In the next section, we consider general patterns and implications of these results.

The participants’ scaled scores on the DAYC-2 were at least in the average range (in fact, over 90) on both scales on all three observations. This assessment indicates no concerns about either general cognitive or social–emotional development for these participants.

Most of the scores on the BRIEF-P, including all of the Global Executive Composite scores except for one, remain below the cutoff for potential clinical concern (65). The group means were not at clinical risk for any Index or GEC. Many of the score changes over time for Nayla and Haven are large enough to be considered reliable, according to the test–retest reliability table provided in the scoring manual (see also Sherman & Brooks, 2010). One participant (Nayla) had elevated risk scores on EMI and GEC at T1, but decreased to typical levels at T2 and T3. One participant (Haven) had elevated risk scores on ISCI and EMI at T1, and on ISCI and FI at T3. As ISCI and FI have in common ‘Emotional Control’, this may be an area of continuing concern. We note, however, that Haven’s GEC scores never exceeded the threshold of 65.

The short form of the ASL-CDI that we developed has not been normed or tested with a comparison group. However, Caselli et al. (2020) compared the proportion of items known by participants in the full version with randomly sampled subsets of items. Even with 30 items, both receptive and expressive scores are highly correlated with the full test. For this reason, we interpret our results by comparing the scores of our participants to the mean, upper end, and lower end values provided by Caselli et al. for each age. On all three assessments, Ellie’s score was within or above this band, and Nayla’s was within. However, Haven’s scores are consistently below the band. Over time, the distance between her score and the lower end decreases, for both receptive and expressive scores. That is, her increase is more than what would be expected by age alone.

For each child, the number of words known on the English CDI increases over time. The English CDI norms come from typically hearing, monolingual participants, and are only available until the age of 30 months. For this reason, we take comparisons with norm percentile results with caution. With this in mind, we observe that by Time 2, the scores of all three case study participants are within 1 standard deviation of the mean for 30-month-olds, and these scores increase at Time 3.

All three of the Family ASL participants achieved standard scores on the ASL-RST above 100 for the 4-year-old group. As Haven is close to age 5 at the time of testing, we converted her score to a standard score for a 5-year-old, yielding a score of 101. Based on these comparisons, we conclude that the three Family ASL participants are at or above expectations for their age on this assessment.

Each of the participants achieved a score in the ‘average’ range for their age on the CELF-P3, when compared with monolingual, typically hearing English-speaking children.

## 9. General Discussion and Conclusions

Our study began with an expectation that if language assessments (ASL and/or English) are within or close to expectations for age, then the non-language assessments should also display age-appropriate levels. In the following paragraphs, we summarize the participants’ language performance; then, we turn to their performance on the non-language assessments. Before we dive in, we remind the reader how these three participants fare when considering factors commonly associated with higher language levels in DHH children (e.g., Ching et al., 2018; Cupples et al., 2018). All three children are profoundly deaf pre-aided. Children with profound hearing levels are more likely to experience language delays than children with moderate or severe levels. However, none of the three children had an additional disability, and all of the children had highly educated mothers—these are factors that tend to be associated with better outcomes. A major factor associated with better outcomes is age at hearing device fitting. Haven’s CIs were activated at 12 months, Ellie’s at 20 months, and Nayla’s at 26 months. Pre-implant activation, Ellie used bilateral hearing aids from 7 to 9 months, but had relatively little access to speech between 9 and 20 months. Nayla’s early experience with hearing aids was uncertain until the age of 9 months, and her CI activation was the latest of the three. Haven’s enhanced access to speech was the earliest of the three, with CI activation at 12 months.

The three case studies reported here display different patterns in terms of development of ASL and English, and we can already see some patterns that may be related to parental input in ASL and spoken English. Ellie’s ASL input appears to be very strong, given her mother’s ASL-CDI scores and the proportion of signed or bimodal utterances in her input language sample. In addition, she receives substantial spoken English input, with CI activation at 20 months. Accordingly, she shows strengths in both ASL and English, consistently achieving the highest scores of the three children. Her scores for the ASL measures are especially striking; even at the beginning of the longitudinal period she had a high score on ASL vocabulary, and by the end of the study, she achieved ceiling performance on both ASL and English vocabulary, with standard scores above 100 on overall measures for both languages. Note also that her mother started to learn ASL when Ellie was only 2 months old and has used it consistently with her ever since (mostly in bimodal format, with speech and signs together).

Nayla’s ASL input is also strong: her mother achieved high ASL-CDI scores and produced a notable number of sign-only utterances in some of the language samples analyzed; however, this varied quite a bit, and some samples had very small proportions of sign (or bimodal) productions. Nayla’s access to spoken English using CIs was the latest of the three children, at 26 months. Nayla’s own profile shows relative strength in ASL, and her English is also developing within the typical range. Both Nayla and her mother produce a larger proportion of speech-only utterances than Ellie and her mother, but signing is still very much in evidence, often produced without simultaneous speech. These modality results are consistent with Nayla’s scores on ASL vocabulary, which are consistently near the upper end of the observations made in the norming study. Her scores on English vocabulary are lower, but still within the range of 1 standard deviation below the mean, keeping in mind that norms are only available for children up to the age of 30 months. This puts Nayla with the higher-performing children in the cohort examined by Pontecorvo et al. (2023). By the end of our study, Nayla achieves standard scores above 100 on both overall measures.

Finally, Haven’s ASL input is much less consistent than that of the other two participants, and her English access was the earliest, with CI activation at 12 months. Her mother typically used almost exclusively spoken English in our modality analysis, with one notable exception at age 3;11 (Haven’s mother chose to use almost all ASL in that session on her own; we did not ask her to change her performance). Haven’s mother also had lower scores than the other two on the ASL-CDI. In parallel to this, Haven’s profile shows relative strength in English. Her scores on English vocabulary are high, nearly reaching ceiling by the end of the study. In contrast, her ASL scores tend to be lower than those of the other two children, despite the fact that she is the oldest of the three. Nevertheless, we see evidence that Haven’s ASL appears to be progressing. Her score for ASL vocabulary does not reach the lower-end band for age-mates found by Caselli et al. (2020) for deaf children with deaf parents, but she is moving toward that level and closing the gap across the three observations. Like Ellie and Nayla, Haven also achieved standard scores above 100 on both overall measures.

We consider all three participating children to be developing as bimodal bilinguals. Like other bilingual children, they show varying levels of proficiency in each of their languages. While operationalization of the notion of ‘dominance’ is complex (Treffers-Daller & Silva-Corvalán, 2016), we would roughly characterize Ellie and Nayla as relatively balanced in ASL and English, and Haven as dominant in spoken English. Such a characterization is compatible with both the estimated relative amount of exposure each child experiences with the two languages, and their own developmental trajectories.

Given the overall language strengths for all three children, we are not surprised to see that they all have generally good scores on the non-language tasks. Their scores are above 90 on both the cognitive and the social–emotional domains of the DAYC-2 on all three observations, and they exceed 100 on most administrations.

On the BRIEF-P, we used the cutoff of 65 as an indicator of potential clinical concern. Nayla’s Global Executive Composite, at 68, did exceed this cutoff at Time 1, but decreased to typical levels at Time 2 and Time 3. Neither Ellie nor Haven had GEC scores above 65 on any administration. However, Haven did have elevated risk scores on ISCI and EMI at T1, and on ISCI and FI at T3. As ISCI and FI both include a measure of ‘Emotional Control’, this may be an area of continuing concern for Haven.

It is important to remember that these three individual children and their families should not be taken as representative of DHH children as a whole. As mentioned earlier, developmental outcomes for deaf children in hearing families are highly variable, likely reflecting wide variation in their access to early language input. In contrast, the children in the current study all came from families that benefited from the information and resources to commit to a bilingual approach that included ASL and spoken English, either at home or at preschool. As a result, Ellie, Nayla and Haven experienced access to a sign language and/or spoken language by the age of 12 months. Their families were highly motivated to support their DHH children’s development, taking advantage of ASL classes, ASL tutors, and even (for Nayla and Haven) seeking out signing preschool programs to increase their children’s signed input. Relatively few families have the resources to provide this level of sign language support for their DHH children.

Nevertheless, these 3 case studies paint a picture of what it is possible for hearing families with DHH children to achieve. They also demonstrate that, like other bilingual child populations, language dominance varies across individuals despite access to more than one language. This includes children who achieve high levels of performance in both languages; some may excel in English, with ASL developing as a strong secondary language, and yet others may excel in ASL, with English developing into a strong secondary language. Whatever the child’s individual pattern of language dominance, either language can serve as a strong foundation on which other skills may be built.

Overall, we see no evidence that early introduction of a sign language will obstruct DHH children’s development of a spoken language. Although our sample number is small, all 3 families expressed strong motivation for learning and using ASL with their DHH child. Crucially, the DHH children we observed achieved positive language and cognitive development outcomes even while their parents were relatively new signers, countering the myth that hearing parents’ non-fluent ASL input precludes their DHH children from successfully developing as ASL signers. By partnering with Deaf adults as additional sources of ASL input for their children (through Deaf mentors, preschool programs, ASL story time, etc.), novice signer parents can support their children’s development as ASL-English bilinguals, establishing a strong foundation for further cognitive and linguistic growth.

## Figures and Tables

**Table 1:** Participant information.

Child	Chronological Age over Observation Period	Sex	Race	Mother’s Education	Siblings	Pre-Aided Hearing Level	Hearing Technology (Age at Activation)
Ellie	2;00–4;04	F	White	Completed undergraduate degree	2 older (hearing)	Profound	Bilateral CI (1;08)
Nayla	2;04–4;03	F	White	Completed undergraduate degree	1 older (hearing)	Profound	Bilateral CI (2;02)
Haven	3;02–5;00	F	White	Graduate degree	1 older (hearing)	Profound	Bilateral CI (1;00)

**Table 2:** Results from administration of the American Sign Language Communicative Development Inventory (ASL-CDI) (Caselli et al., 2020), 100-item version, to each child’s participating parent. The child’s age at the point when the assessment was completed is provided. Scores are expressed as number of items out of 100.

	Time 1			Time 2			Time 3		
Mother of	Age	Receptive	Expressive	Age	Receptive	Expressive	Age	Receptive	Expressive
Ellie	2;02	100	100	2;08	100	100	3;00	100	100
Nayla	2;07	95	93	3;01	100	95	3;04	99	98
Haven	3;04	86	69	3;09	84	72	4;01	90	76

**Table 3:** Results from administration of Developmental Assessment of Young Children-2 (DAYC-2) (Voress et al., 2012): Cognitive (Cog) and DAYC-2: Social–Emotional (Soc-Emo) scale expressed as standard scores. Age is given in years;months.days format.

	Time 1			Time 2			Time 3		
Child	Age	Cog	Soc-Emo	Age	Cog	Soc-Emo	Age	Cog	Soc-Emo
Ellie	2;05.12	113	130	2;11.5	116	124	4;00.27	94	107
Nayla	2;09.15	103	108	3;03.25	109	108	4;03.18	95	97
Haven	3;07.3	113	103	4;00.19	108	104	5;00.14	104	105

**Table 4:** Results from administration of the Behavioral Rating Inventory of Executive Function-Preschool Version (BRIEF-P) (Gioia et al., 2003) expressed as T-scores.

	Time 1					Time 2					Time 3				
Child	Age	ISCI	FI	EMI	GEC	Age	ISCI	FI	EMI	GEC	Age	ISCI	FI	EMI	GEC
Ellie	2;05.12	48	41	50	47	2;10.28	54	38	54	52	4;01.5	55	42	43	48
Nayla	2;09.15	63	47	79	68	3;03.19	48	36	54	48	4;00.28	50	45	38	42
Haven	3;06.26	66	52	67	64	4;00.10	49	45	40	42	5;00.3	67	68	48	59

**Table 5:** Results from administration of the American Sign Language Communicative Development Inventory (ASL-CDI) (Caselli et al., 2020), 100-item version, expressed as number of items out of 100. * indicates that the score is proportionally at or above the mean for participants of the same age in the full version of the assessment.

	Time 1			Time 2			Time 3		
Child	Age	Receptive	Expressive	Age	Receptive	Expressive	Age	Receptive	Expressive
Ellie	2;02	99 *	81 *	2;08	100 *	99 *	3;00	100 *	99 *
Nayla	2;07	87 *	71 *	3;01	94 *	74 *	3;04	97 *	80 *
Haven	3;04	39	7	3;09	60	23	4;01	72	35

**Table 6:** Results from administration of MacArthur Bates English Communicative Development Inventory short form (CDI) (Fenson et al., 2000), expressed as number of words known out of 100.

	Time 1		Time 2		Time 3	
Child	Age	Words Known	Age	Words Known	Age	Words Known
Ellie	2;03.3	47	2;08.19	86	3;00.14	100
Nayla	2;06.23	40	3;00.9	71	3;04.1	80
Haven	3;04.10	84	3;09.25	80	4;01.10	97

**Table 7:** Results from administration of the ASL Receptive Skills Test (RST) (Enns et al., 2013), expressed as raw accuracy scores and standard scores.

Child	Age	Raw Score	Standard Score
Ellie	4;01.10	24	119
Nayla	4;01.8	19	112
Haven	4;09.24	16	108

**Table 8:** Results from administration of the Clinical Evaluation of Language Fundamentals Preschool-3 (CELF-P3) (Wiig et al., 2020), expressed as Core Language Standard Scores.

Child	Age	Core Language Standard Score
Ellie	4;01	101
Nayla	4;02	106
Haven	4;11	106

## Data Availability

The original contributions presented in this study are included in the article. Further inquiries can be directed to the corresponding author.

## Notes

1 Visual inspection of the histogram indicates that over 30% of participants scored more than 2 standard deviations below the mean.

2 Commonly cited estimates for this number in the U.S. range between 10 and 30%, but these figures are not supported by current research. The last national survey of DHH children conducted by the Gallaudet Research Institute reported on data from 2013 to 2014 (Gallaudet Research Institute, 2014). This survey reported that ASL was used in the homes of 13.2% of DHH children. We know of only one recent quantitative survey in the US. Jones and Roberts (2024) surveyed 105 caregivers of 12–18-month-old DHH children, and found that 63.8% responded positively to, “I use sign with my child and/or others use sign with my child.” Figures are lower on surveys in other countries. Crowe et al. (2014) found that 20.9% of the 177 Australian caregivers they surveyed reported that sign was used with their child, but in a more recent and larger study, Howell et al. (2024) found that 8.7% of the 997 parents in their Australian survey reported using Auslan (Australian Sign Language) at home.

3 It should be borne in mind that scores on ‘non-verbal’ measures are often related to language experience (Quam & Coppola, 2023).
